# Apoptosis Induced by the Curcumin Analogue EF-24 Is Neither Mediated by Oxidative Stress-Related Mechanisms nor Affected by Expression of Main Drug Transporters ABCB1 and ABCG2 in Human Leukemia Cells

**DOI:** 10.3390/ijms18112289

**Published:** 2017-10-31

**Authors:** Nikola Skoupa, Petr Dolezel, Eliska Ruzickova, Petr Mlejnek

**Affiliations:** Department of Anatomy, Faculty of Medicine and Dentistry, Palacky University Olomouc, Hnevotinska 3, Olomouc 77515, Czech Republic; NikolaSkoupa@seznam.cz (N.S.); p.dolezel@atlas.cz (P.D.); elis.ruzickova@gmail.com (E.R.)

**Keywords:** NF-κB, Nrf2, EF-24-GSH adduct, EF-24-NAC adduct, K562 cells

## Abstract

The synthetic curcumin analogue, 3,5-bis[(2-fluorophenyl)methylene]-4-piperidinone (EF-24), suppresses NF-κB activity and exhibits antiproliferative effects against a variety of cancer cells in vitro. Recently, it was reported that EF-24-induced apoptosis was mediated by a redox-dependent mechanism. Here, we studied the effects of *N*-acetylcysteine (NAC) on EF-24-induced cell death. We also addressed the question of whether the main drug transporters, ABCB1 and ABCG2, affect the cytotoxic of EF-24. We observed that EF-24 induced cell death with apoptotic hallmarks in human leukemia K562 cells. Importantly, the loss of cell viability was preceded by production of reactive oxygen species (ROS), and by a decrease of reduced glutathione (GSH). However, neither ROS production nor the decrease in GSH predominantly contributed to the EF-24-induced cell death. We found that EF-24 formed an adduct with GSH, which is likely the mechanism contributing to the decrease of GSH. Although NAC abrogated ROS production, decreased GSH and prevented cell death, its protective effect was mainly due to a rapid conversion of intra- and extra-cellular EF-24 into the EF-24-NAC adduct without cytotoxic effects. Furthermore, we found that neither overexpression of ABCB1 nor ABCG2 reduced the antiproliferative effects of EF-24. In conclusion, a redox-dependent-mediated mechanism only marginally contributes to the EF-24-induced apoptosis in K562 cells. The main mechanism of NAC protection against EF-24-induced apoptosis is conversion of cytotoxic EF-24 into the noncytotoxic EF-24-NAC adduct. Neither ABCB1 nor ABCG2 mediated resistance to EF-24.

## 1. Introduction

Many polyphenolic compounds extracted from plants have been demonstrated to have cancer-preventing activities in laboratory studies. Curcumin, a component of turmeric (*Curcuma longa*), is a typical example. This agent was reported to inhibit proliferation and survival of cancer cells in vitro, while retaining a pharmacologically safety profile in vivo. However, clinical studies show that curcumin has low efficacy due to rapid excretion in vivo [1,2]. This prompted the development of analogues, which are more potent inducers of apoptosis in in vitro assays and also more efficient in vivo.

Adams and co-workers synthesized 3,5-bis[(2-fluorophenyl)methylene]-4-piperidinone (EF-24), a fluorinated curcumin analog which exhibits antiprolifative effects against a variety of cancer cells in vitro [3,4,5,6,7,8,9] and also in animal models in vivo [10,11,12]. Unlike curcumin, EF-24 is bioavailable with a greater potency to induce cell death in many tumors and shows relatively low toxicity [13]. It does this through direct inhibition of IκB kinase (IKK), resulting in suppression of NF-κB activity [5,12]. In addition, the antiproliferative effects of EF-24 may also be mediated by deregulation of important signaling pathways that include upregulation of PTEN, downregulation of Akt or inhibition HIF-1α in certain cancer cell lines in vitro [7,8,9,10]. Several reports also suggest that the anticancer activity of EF-24 may be mediated, in part, by a redox-dependent induction of apoptosis [14,15] or due to increased reactive oxygen species (ROS) production [9,16,17].

Here we address the question of how much oxidative stress contributes to the cell death induction in EF-24-treated human leukemia K562 cells. We also studied whether ABCB1 (P-glycoprotein) and ABCG2 (BCRP, breast cancer resistance protein), which are usually considered the main drug transporters underlying the multidrug resistance (MDR) phenotype in cancer cells, compromise the antiproliferative effects of EF-24 in K562 cells. We observed that ROS production and glutathione (GSH) depletion contributed to EF-24-induced cell death only slightly. Importantly, neither ABCB1 nor ABCG2 expression reduced the cytotoxic effects of EF-24.

## 2. Results

### 2.1. N-Acetylcysteine (NAC) Prevented 3,5-bis[(2-Fluorophenyl)Methylene]-4-Piperidinone (EF-24)-Induced Cell Death with Apoptotic Hallmarks in K562 Cells

**Figure 1:**
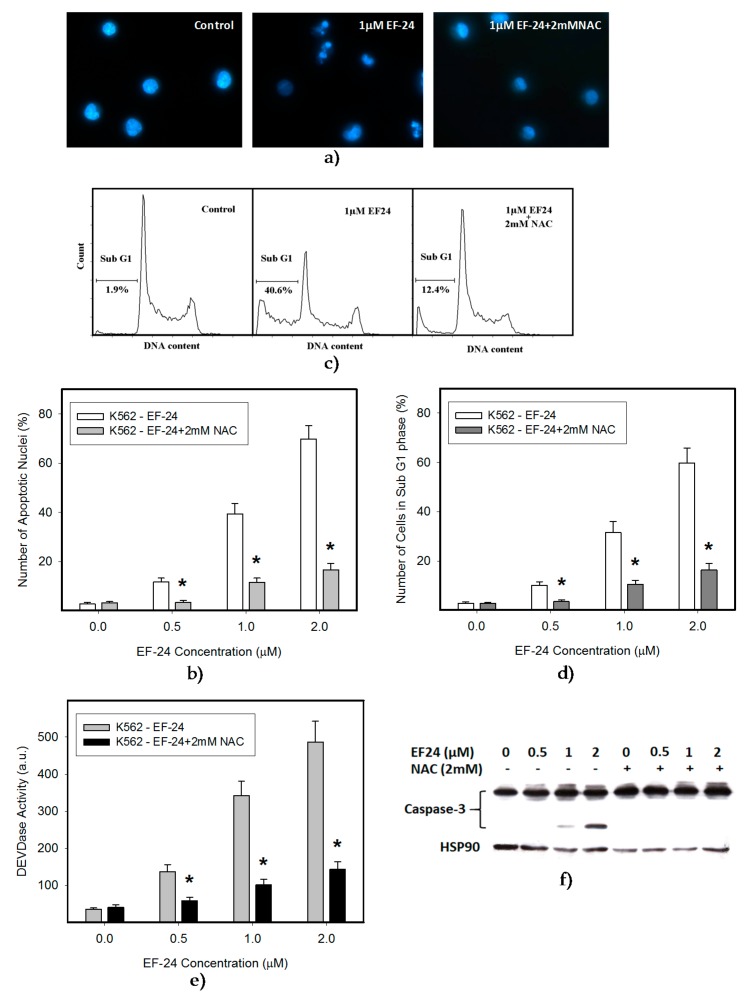
Effect of *N*-acetylcysteine (NAC) on cell death in 3,5-bis[(2-fluorophenyl)methylene]-4-piperidinone (EF-24)-treated cells. Cells were treated with EF-24 or with EF-24 + 2 mM NAC, as indicated. Incubation proceeded for 24 h prior to appropriate analysis. The experimental points represent mean values from three replicate experiments, with standard deviations. (**a**) Effect of NAC on nuclear morphology in EF-24 treated cells. Pictures represent typical examples. (**b**) A quantitative analysis of the effect of NAC on nuclear morphology in EF-24 treated cells. At least 300 cells were examined in each experiment; * denotes significant change in the number of cells with apoptotic nuclei (*p* < 0.05) between K562 cells treated with EF-24 and cells treated with EF-24 + NAC. (**c**) Effect of NAC on occurrence of cells in sub G1 phase in response to EF-24 treatment. A representative analysis. (**d**) A quantitative analysis of the effect of NAC on occurrence of cells in sub G1 phase in response to EF-24 treatment; * denotes significant change in the number of cells sub G1 phase (*p* < 0.05) between K562 cells treated with EF-24 and cells treated with EF-24 + NAC. (**e**) Effect of NAC on caspase-3 activation in EF-24-treated cells. After 16 h, cells DEVDase enzymatic activity was determined in cell lysates using Ac-DEVD-AMC; * denotes significant change in DEVDase enzymatic activity (*p* < 0.05) between K562 cells treated with EF-24 and cells treated with EF-24 + NAC. (**f**) Effect of NAC on caspase-3 processing in EF-24 treated cells. After 16 h, caspase-3 processing was monitored using western blot analysis. Picture represents a typical example.

**Figure 2:**
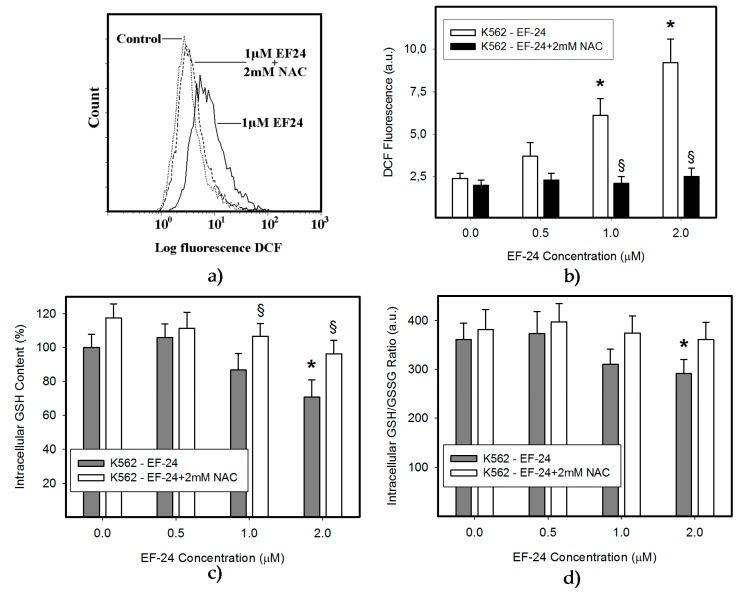
Effect of NAC on EF-24-induced oxidative stress. Cells were treated with EF-24 or with EF-24 + 2 mM NAC, as indicated. The experimental points represent mean values from three replicate experiments, with standard deviations. (**a**) Effect of NAC on reactive oxygen species (ROS) production in EF-24-treated cells. ROS production was determined using flow cytometry after 3 h incubation. A representative analysis. (**b**) A quantitative analysis of the effect of NAC on ROS production in response to EF-24 treatment. ROS production was determined using flow cytometry after 3 h incubation; * denotes significant change in ROS production (*p* < 0.05) between control (untreated) K562 cells and cells treated with EF-24; § denotes significant change in ROS production (*p* < 0.05) between EF-24 treated K562 cells and cells treated with EF-24 + NAC. (**c**) Effect of NAC on intracellular glutathione (GSH) level in EF-24 treated cells. After 3 h incubation, the intracellular content of GSH was determined using LC/MS/MS analysis; * denotes significant change in the intracellular level of GSH (*p* < 0.05) between control (untreated) K562 cells and cells treated with EF-24; § denotes significant change in intracellular level of GSH (*p* < 0.05) between K562 cells treated with EF-24 and cells treated with EF-24 + NAC. (**d**) Effect of NAC on GSH/oxidized glutathione (GSSG) ratio in EF-24-treated cells. After 3 h incubation, the intracellular content of GSH and GSSG were determined using LC/MS/MS analysis; * denotes significant change in GSH/GSSG ratio (*p* < 0.05) between K562 cells treated with EF-24 and cells treated with EF-24 + NAC.

**Figure 3:**
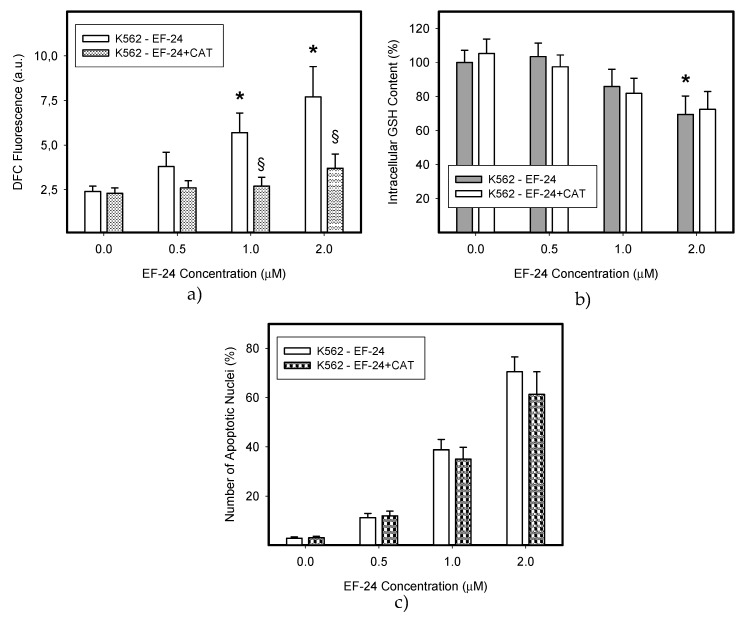
Effect of catalase (CAT) on EF-24-induced oxidative stress. Cells were treated with EF-24 or with EF-24 + CAT (50 U/mL), as indicated. The experimental points represent mean values from three replicate experiments, with standard deviations. (**a**) Effect of CAT on ROS production in EF-24 treated cells. After 3 h incubation, ROS production was determined using flow cytometry; * denotes significant change in ROS production (*p* < 0.05) between control (untreated) K562 cells and cells treated with EF-24; § denotes significant change in ROS production (*p* < 0.05) between EF-24-treated K562 cells and cells treated with EF-24 + CAT. (**b**) Effect of CAT on intracellular GSH level in EF-24 treated cells. After 6 h incubation, the intracellular content of GSH was determined using LC/MS/MS analysis; * denotes significant change in intracellular level of GSH (*p* < 0.05) between control (untreated) K562 cells and cells treated with EF-24. (**c**) Effect of CAT on nuclear morphology in EF-24-treated cells. After 24 h, cells were stained using Hoechst33342, and nuclear morphology was examined using fluorescence microscopy.

In this study we used human leukemia K562 cells, since they represent a type of malignancy with impaired NF-κB activity [18]. We observed that EF-24 induced cell death with apoptotic hallmarks, including chromatin condensation, DNA fragmentation and caspase-3 activation (Figure 1, Table 1). Importantly, EF-24-induced cell death was preceded by ROS production, which was significant but moderate (Figure 2a,b), and by the reduction of GSH levels within K562 cells (Figure 2c). Despite the fact that enhanced ROS production and the drop in GSH levels might indicate oxidative stress, an increased level of oxidized glutathione (GSSG) was not found as the GSH/GSSG ratio was not dramatically changed (Figure 2d). Addition of *N*-acetylcysteine (NAC) at millimolar concentrations into the growth medium abrogated ROS production (Figure 2a,b), GSH depletion (Figure 2c) and prevented apoptosis induction in EF-24-treated K562 cells (Figure 1, Table 1). Interestingly, pretreatment of cells with NAC at sub-milimolar concentrations diminished ROS production, restored GSH levels but, it prevented cell death only partially (not shown). In contrast, addition of catalase (CAT) into the growth medium abrogated ROS production (Figure 3a), but it did not prevent GSH depletion (Figure 3b), and failed to reduced cell death (Figure 3c, Table 1).

### 2.2. EF-24 Did Not Activate Nuclear Factor-Erythroid 2 Related Factor 2 (Nrf2) in K562 Cells

**Figure 4:**
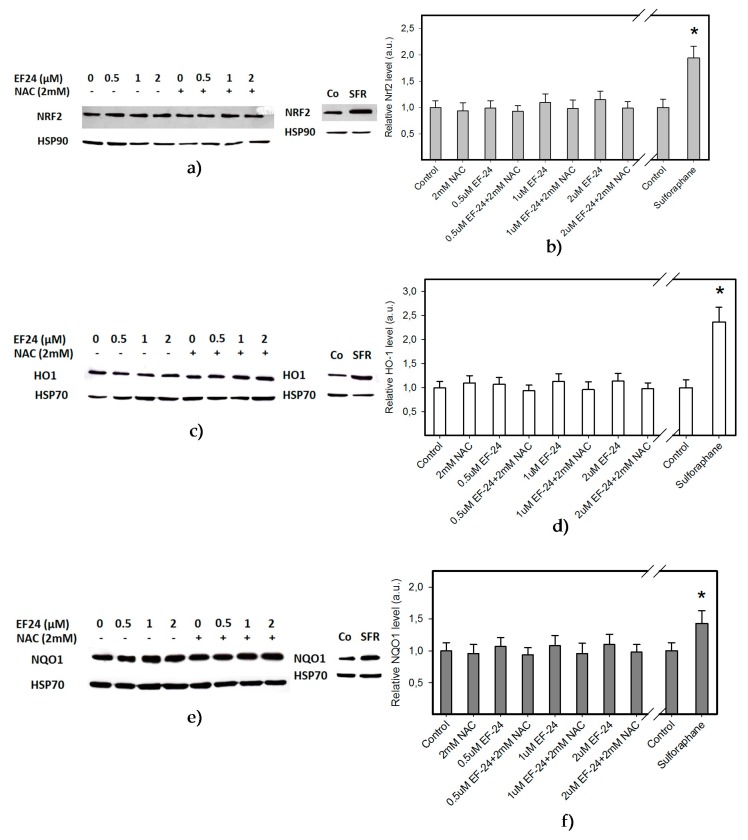
Effect of EF-24 on the activation of Nrf2 regulated signaling pathway. Cells were treated with EF-24 or with EF-24 + 2 mM NAC, as indicated. Incubation proceeded for 6 h prior to Western blot analysis. Treatment of cells with sulforaphane was used as a positive control [21]. (**a**) Effect of EF-24 on the activation of Nrf2. A representative example. (**b**) A quantitative analysis of the effect of EF-24 on the activation of Nrf2; * denotes significant change in the Nrf2 level (*p* < 0.05) between untreated K562 cells and cells treated with sulforaphane. (**c**) Effect of EF-24 on the activation of heme oxygenase-1 (HO-1). A representative example. (**d**) A quantitative analysis of the effect of EF-24 on the activation of HO-1; * denotes significant change in the HO-1 level (*p* < 0.05) between untreated K562 cells and cells treated with sulforaphane. (**e**) Effect of EF-24 on the activation of NAD(P)H:quinon oxidoreductase 1 (NQO1). A representative example. (**f**) A quantitative analysis of the effect of EF-24 on the activation of NQO1; * denotes significant change in NQO1 levels (*p* < 0.05) between untreated K562 cells and cells treated with sulforaphane.

To evaluate the significance of ROS production in the fate of the cell, we addressed the question of whether EF-24 is capable of activating the nuclear factor-erythroid 2 related factor 2 (Nrf2). It is well documented that cells respond to oxidative stress by activation of cellular defense mechanisms that comprise several antioxidant pathways regulated by Nrf2 [19,20]. However, our results clearly indicated that EF-24 did not activate the Nrf2 signaling pathway. Indeed, neither increased levels of Nrf2 nor elevated expression levels of stress-response proteins, including heme oxygenase-1 (HO-1) and NAD(P)H:quinon oxidoreductase 1 (NQO1) were observed in EF-24-treated K562 cells (Figure 4). These results, together with those described in the previous paragraph, collectively suggest that increased production of ROS played only a marginal role in the induction of the death of EF-24-treated K562 cells.

### 2.3. Conversion of Cytotoxic EF-24 Into the Non-Cytotoxic EF-24-NAC Adduct is the Main Mechanism of the NAC Protection Against the Cytotoxic Effects of EF-24

**Figure 5:**
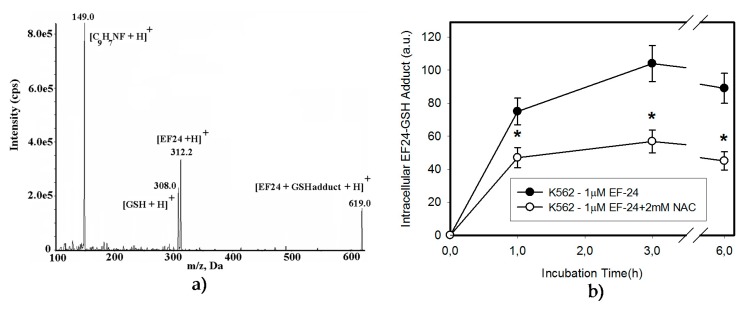
EF-24 forms adduct with GSH. (**a**) MS/MS analysis of the EF-24-GSH adduct: Mass spectrum of the molecular ion of EF-24-GSH adduct (m/z 619.0), result fragment ion [EF-24 + H]^+^, m/z 312.2, [GSH + H]^+^, m/z 308.0, and [C9H7NF+H]^+^, *m*/*z* 149.0. (**b**) Effect of NAC on EF-24-GSH adduct formation. Cells were treated with EF-24 or in the combination with 2 mM NAC for given incubation times. The amount of the EF-24-GSH adduct in cells was determined using LC/MS/MS. The experimental points represent mean values from three replicate experiments, with standard deviations; * denotes significant change in EF-24-GSH adduct content between EF-24 and EF-24 + NAC-treated K562 cells.

**Figure 6:**
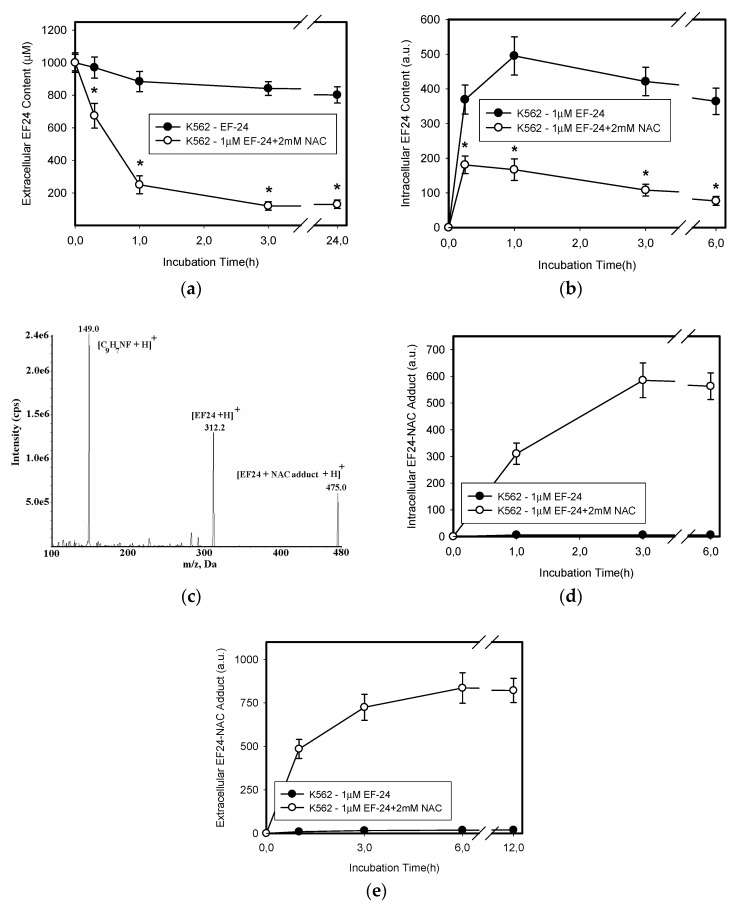
Effect of NAC on extracellular and intracellular levels of EF-24. Cells were treated with EF-24 or with EF-24 + 2 mM NAC. At indicated time intervals, extracellular and intracellular levels of EF-24 were determined using LC/MS/MS analysis. (**a**) Extracellular EF-24 level; * denotes significant change in EF-24 content in growth medium (*p* < 0.05) between EF-24 and EF-24 + NAC-treated K562 cells. (**b**) Intracellular EF-24 level; * denotes significant change in intracellular EF-24 content (*p* < 0.05) between EF-24 and EF-24 + NAC-treated K562 cells. The experimental points represent mean values from three replicate experiments, with standard deviations. (**c**) MS/MS analysis of EF-24-NAC adduct: Mass spectrum of the molecular ion of [EF-24-NAC-adduct]^+^, m/z 475.0, result fragment ion [EF-24+H]^+^, m/z 312.2, and [C9H7NF + H]^+^, m/z 149.0. (**d**) Time course of intracellular EF-24-NAC adduct formation. Cells were treated with EF-24 or with EF-24 + NAC for given incubation time. The amount of EF-24-NAC adduct in cells was determined using LC/MS/MS. (**e**) Time–course of extracellular EF-24-NAC adduct formation. Cells were treated with EF-24 or with EF-24 + NAC for given incubation times. The amount of EF-24-NAC adduct in growth medium was determined using LC/MS/MS. The experimental points represent mean values from three replicate experiments, with standard deviations.

Excessive oxidation of GSH as a result of oxidative stress, or its conjugation with xenobiotics represent important mechanisms that may lead to the depletion of cellular GSH [22,23]. Given that no increased level of GSSG was found in cells (Figure 2d) or culture medium (not shown), we studied the interaction of EF-24 and GSH in more detail. As expected, we found intracellular formation of the EF-24-GSH adduct in K562 cells (Figure 5a,b). The amount of EF-24-GSH adduct was proportional to the concentration of EF-24 (not shown). Formation of the EF-24-GSH adduct was significantly reduced by the addition of NAC (Figure 5b). While GSH depletion seemed to be related to the EF-24-GSH adduct formation, the restoration of GSH synthesis using NAC could not fully explain the mechanism of NAC protection against EF-24-induced apoptosis. Indeed, we found that adding NAC to the culture medium induced a rapid intra- and extra-cellular clearance of EF-24 (Figure 6a,b). This was accompanied by intra- and extra-cellular formation of the EF-24 adduct with NAC (Figure 6c–e). These results clearly indicated that conversion of cytotoxic EF-24 into the non-cytotoxic EF-24-NAC adduct was the main mechanism of NAC protection. It is important to point out that EF-24 formed mono- and di-adducts with NAC and/or GSH, however, the mono-adducts were predominant (not shown). For this reason, the data presented here refer to the appropriate mono-adducts.

**Figure 7:**
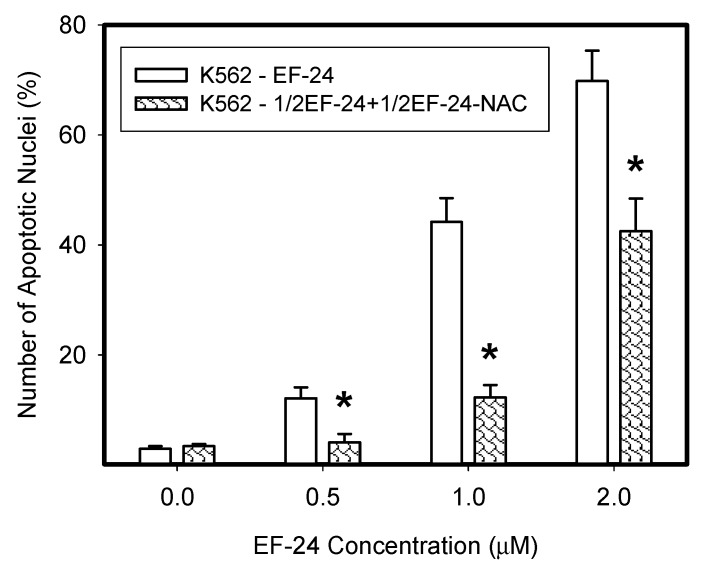
Proapoptotic effects of the EF-24-NAC adduct. EF-24 (50 μM) was mixed with 2 mM NAC in distilled water at ambient temperature and reaction was monitored using LC/MS/MS analysis. When 50% of EF-24 was converted into the EF-24-NAC adduct, reaction was stopped by dilution. Then K562 cells were treated with EF-24 alone or with diluted reaction mixture containing the same amount of EF-24, however, approximately 50% of it was converted into EF-24-NAC adduct. The experimental points represent mean values from three replicate experiments, with standard deviations; * denotes significant change in the number of cells with apoptotic nuclei (*p* < 0.05) between K562 cells treated with “free” EF-24 and cells treated with a diluted mixture containing the same amount of EF-24 (50% “free” EF-24 + 50% EF-24-NAC adduct).

To directly demonstrate that the EF-24-NAC adduct was not cytotoxic, we used a mixture of EF-24 and NAC in distilled water, where the reaction was allowed to proceed until 50% of EF-24 was converted into the EF-24-NAC adduct (not shown). K562 cells were then treated with the diluted reaction mixture. We observed that the proapoptotic effect of the reaction mixture, containing approximately one half of remnant free EF-24 and one half of EF-24-NAC adduct, was equivalent to the effect of remnant free EF-24 (Figure 7). For example, a diluted reaction mixture containing approximately 1 μM EF-24 and 1 μM EF-24-NAC adduct exhibited a proapoptotic effect, which was similar to that of 1 μM EF-24 itself rather than to the effect of 2 μM EF-24 (Figure 7).

### 2.4. Overexpression of ABCB1 and ABCG2 Did Not Compromise the Antiproliferative Effects of EF-24 in K562 Cells

**Figure 8:**
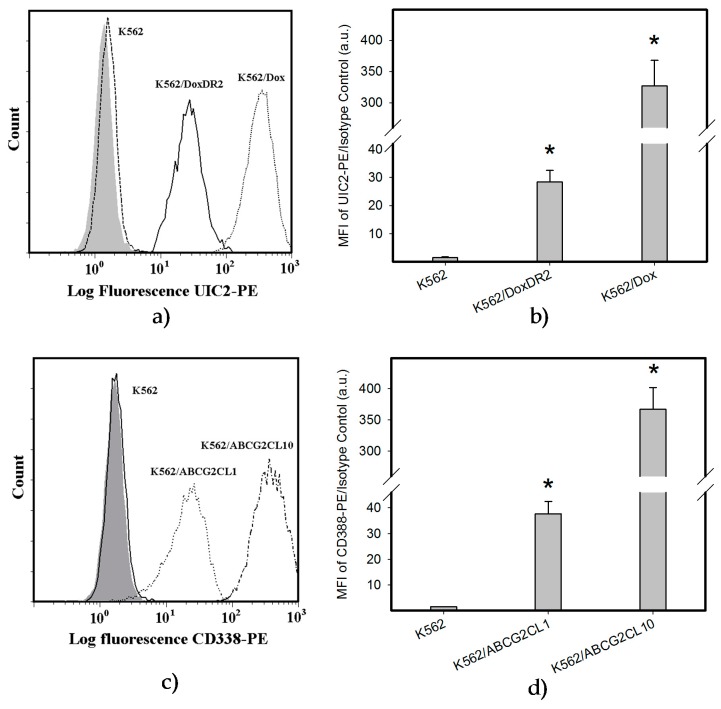
Analysis of ABCB1 and ABCG2 expression: (**a**) Flow cytometric analysis of ABCB1 expression. Isotype control (grey histogram); K562 parental cell line (dash line); K562/Dox cells (dot line); K562/DoxDR2 cells (solid line). (**b**) Quantitative analysis of ABCB1 expression. ABCB1 expression was quantified as the mean fluorescence intensity (MFI) shift (ratio of MFI of UIC2-PE antibody and isotype control). The experimental points represent mean values from three replicate experiments, with standard deviations; * denotes significant change in ABCB1 expression (*p* < 0.05) between K562 cells and cells expressing various levels of ABCB1 (K562/Dox, K562/DoxDR2). (**c**) Flow cytometric analysis of ABCG2 expression. Isotype control (grey histogram); K562 parental cell line (solid line); K562/ABCG2CL10 cells (dash-dot line); K562/ABCGCL1 cells (dot line). (**d**) Quantitative analysis of ABCG2 expression. ABCG2 expression was quantified as the mean fluorescence intensity (MFI) shift (ratio of MFI of CD338-PE antibody and isotype control). The experimental points represent mean values from three replicate experiments, with standard deviations; * denotes significant change in ABCG2 expression (*p* < 0.05) between K562 cells and cells expressing various levels of ABCG2 (K562/ABCGCL10, K562/ABCGCL1).

**Figure 9:**
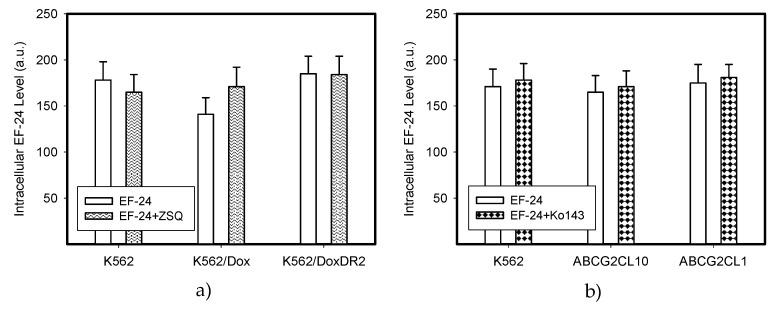
Intracellular levels of EF-24 in cells expressing ABCB1 or ABCG2. The parental cell line K562, which does not express any of the transporters was used as a control. (**a**) Relative intracellular levels of EF-24 in cells overexpressing ABCB1 transporter. Cells were treated with EF-24 or with EF-24 + inhibitor zosuquidar trihydrochloride (ZSQ), as indicated. The experimental points represent mean values from three replicate experiments, with standard deviations. (**b**) Relative intracellular levels of EF-24 in cells overexpressing ABCG2 transporter. Cells were treated with EF-24 or with EF-24 + inhibitor Ko143, as indicated. The experimental points represent mean values from three replicate experiments, with standard deviations.

Decreased intracellular drug levels, which prevent effective interaction between the drug and its cellular target, is a generally accepted mechanism of resistance mediated by ABC transporters [24,25]. Therefore, we analyzed extracts from cells expressing ABCB1 and ABCG2, the main drug transporters (Figure 8 and Figure 9). We found that neither the overexpression of ABCB1 nor ABCG2 significantly reduced the intracellular levels of EF-24 (Figure 9). Accordingly, neither ABCB1 nor ABCG2 expression mediated resistance to EF-24 (Table 2). Importantly, EF-24 exhibited a proapoptotic effect in ABCB1- and ABCG2-expressing cells similar to that found in parental K562 cells (not shown). Our results clearly indicate that EF-24 would exhibit antiproliferative effects, irrespective of ABCB1 or ABCG2 expression.

## 3. Discussion

A large number of studies have demonstrated aberrant NF-κB signaling in solid cancers, as well as in various types of hematologic malignancies. NF-κB is involved in regulating the expression of a number of genes that affect proliferation, cell survival, tumor metastasis, angiogenesis and inflammation. Therefore, targeting aberrant NF-κB activation together with its upstream and downstream interacting regulatory molecules using low molecular weight inhibitors, may be useful in clinical settings for the treatment of solid cancers and hematological malignancies [18].

Our results indicate the strong proapoptotic potential of EF-24 in K562 cells (Figure 1, Table 1). Its proapoptotic effect is much higher than that demonstrated in colorectal or gastric cancer cells [16,17]. This is not surprising, since chronic myelogenous leukemia cells rely on aberrant activation of NF-κB [18], and EF-24 efficiently suppresses NF-κB signaling through direct inhibition of IKK [5,12]. However, several laboratories have shown that the proapoptotic effects of EF-24 are also mediated by a redox-dependent mechanism [14,15,16,17]. A recent publication concluded that EF-24 induces apoptosis via ROS-dependent mitochondrial dysfunction in human colorectal cancer cells [17]. This is supported by the findings that the EF-24-induced cell death was preceded by production of ROS, GSH depletion and mainly by the observation that NAC, a well-known radical scavenger and a precursor of GSH synthesis, prevented cell death [23]. Our results suggest that the EF-24-induced cell death with apoptotic features (Figure 1) was preceded by transient production of ROS (not shown) with a peak at 3 h after the EF-24 addition, and by a decrease in GSH levels in human leukemia K562 cells (Figure 2). However, in contrast to the conclusions published by He and co-workers [17], we do not think that ROS production itself is the direct cause of cell death. This conclusion stems from following findings. First, although CAT prevented ROS production (Figure 3a), it failed to reduce the antiproliferative effects of EF-24 (Table 1, Figure 3c). Second, even though ROS production and reduced intracellular level of GSH are typical signs of oxidative stress, these were not accompanied by elevated levels of GSSG (Figure 2d). In contrast to expectations, we observed that, both GSH and GSSG decreased, however, the decrease in GSSG was somewhat lower than that of GSH for 2 μM EF-24 (Figure 2d). There was no evidence for any other mechanisms of GSH depletion, such as leakage of GSH and/or GSSG into the growth medium (not shown). Third, we also failed to find any signs of Nrf2 activation. Nrf2 is a master regulator of cell response to oxidative stress [19,20]. Under “normal” conditions it is maintained at low levels and resides predominantly in the cytoplasm. Upon oxidative stress, Nrf2 levels increase due to diminished proteasomal degradation, and it translocates to the nucleus to trigger transcription of a plethora of genes to cope with the oxidative stress [19,26]. However, no elevated Nrf2 level (Figure 4a,b) or increased expression of antioxidant genes, including HO-1 (Figure 4c,d) or NQO1 (Figure 4e,f) were found in the present study.

Similarly, the decrease in GSH level itself is probably not a direct cause of cell death. Indeed, EF-24, up to 1 μM concentration, had no effect on inducing a significant decrease in GSH, while it induced significant cell death (Figure 1 and Figure 2). In addition, NAC at sub-millimolar concentrations, affected cell survival only partially, despite significantly reduced the drop in GSH level (not shown).

Instead, EF-24 may serve as a Michael acceptor and form adducts with thiols [15]. Similar to others [15], we found that EF-24 forms mono-adducts (Figure 5) and di-adducts (not shown) with GSH. Since the di-adducts represented only a minor part of the adducts, we analyzed mono-adducts. We hypothesize that the formation of EF-24-GSH adducts may contribute to the decrease in GSH levels (Figure 2c and Figure 3b), since we found no evidence for any other mechanism of GSH depletion, such as leakage of GSH and/or GSSG into the growth medium (not shown).

**Figure 10:**
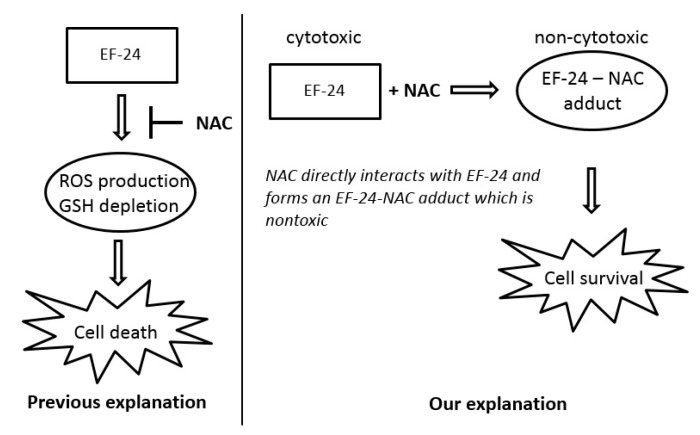
Proposed mechanism of NAC protection against EF-24 cytotoxicity in K562 cells.

NAC prevented the adverse effects of EF-24, including cell death (Figure 1 and Figure 2). However, we do not agree with the interpretation that NAC prevented EF-24-induced cell death due to the abrogation of ROS production and restoration of GSH synthesis [14,15,17]. Our results strongly suggest that addition of NAC at millimolar concentrations into the culture medium induces a rapid conversion of EF-24 into EF-24-NAC adduct (Figure 6) which is non-cytotoxic (Figure 7). Based on our results, we believe that this is the main mechanism of NAC protection against EF-24 cytotoxicity. A summary of our proposed mechanism is shown in Figure 10. It is necessary to note that EF-24 forms mono-adducts (Figure 6c,d) and di-adducts (not shown) with NAC. The di-adducts represented only a minor percentage of adducts. The results presented refer to the mono-adducts for this reason.

We believe that this mechanism of NAC protection plays a crucial role in experimental systems with other cytotoxic agents. For example, cell death induced by geldanamycin (GDN) or by carbonyl cyanide-4-(trifluoromethoxy)phenylhydrazone (FCCP) at high micromolar concentrations is preceded by ROS production and GSH depletion, and can be prevented by NAC addition to the culture medium. In these experimental systems, NAC prevents GDN and FCCP cytotoxicity by conversion of these cytotoxic drugs into corresponding non-cytotoxic adducts with NAC [27,28].

EF-24 may have promise as an anticancer compound applicable in cases of drug resistance, specifically for those which express main drug transporters, ABCB1 and ABCG2 [29]. Indeed, neither ABCB1 nor ABCG2 overexpression significantly reduced the intracellular level of EF-24 in K562 cells (Figure 9). Correspondingly, cells overexpressing ABCB1 or ABCG2 were sensitive to EF-24 in a way that is similar to the parent K562 cells (Table 2). Since drug resistance strongly depends on transporter expression levels [30,31,32,33], we used cells with various expression levels of ABCB1 or ABCG2 in this study. Importantly, cells with high expression levels of drug transporters, which are often used in laboratory experiments, might exhibit a distinct resistance to the studied drug. In contrast, cells expressing lower levels of drug transporters, which may occur in clinical samples [34,35,36], might exhibit a much lower degree of resistance to the particular drug, or the resistance can be completely lost. However, neither moderate nor high expression levels of either transporter failed to mediate resistance to EF-24 in our study (Figure 8 and Figure 9, Table 2). These results indicate that the antiproliferative potential of EF-24 cannot easily be decreased by overexpression of the main drug transporters, ABCB1 and ABCG2, neither in clinical settings nor in the laboratory. In this context, the properties of EF-24 are very similar to those of curcumin, which is not transported by ABCB1 or ABCG2 [37,38].

In conclusion, the results strongly suggest only marginal contribution of a redox-dependent mechanism to EF-24 induced apoptosis in human leukemia K562 cells. The main mechanism of NAC protection against EF-24-induced apoptosis is the conversion of cytotoxic EF-24 into the non-cytotoxic EF-24-NAC adduct. Drug transporters, ABCB1 and ABCG2, do not reduce the antiproliferative effects of EF-24 in K562 cells.

## 4. Material and Methods

### 4.1. Chemicals and Cell Treatment

EF-24 (3,5-bis[(2-fluorophenyl)methylene]-4-piperidinone) and DL-sulforaphane were obtained from Sigma-Aldrich (Saint Louis, MO, USA) and dissolved in DMSO. The residual concentration of DMSO in growth medium was approximately 0.1–0.2%. Catalase (CAT) from bovine liver was dissolved in 25 mM Tris/HCl buffer, pH = 7.0. Cell treatment was done using 50 units of CAT per mL. *N*-acetylcystein (NAC) was dissolved in distilled water. All chemicals were purchased from Sigma-Aldrich (St. Louis, MO, USA). Zosuquidar trihydrochloride (ZSQ; LY335979) and Ko143 (3S,6S,12aS)-1,2,3,4,6,7,12,12a-Octahydro-9-methoxy-6-(2-methylpropyl)-1,4-dioxopyrazino-[1′,2′:1,6] pyrido [3,4-b]indole-3-propanoic acid 1,1-dimethylethyl ester were obtained from Enzo Life Sciences AG (Lausen, Switzerland).

### 4.2. Cell Culture

Human chronic myelogenous leukaemia K562 cells were grown in RPMI-1640 medium supplemented with 10% calf fetal serum and antibiotics in 5% CO_2_ atmosphere at 37 °C. K562 cells were obtained from European Collection of Authenticated Cell Culture (ECACC).

K562/Dox cells overexpressing ABCB1 were kindly provided by Prof J.P. Marie (University of Paris 6, Paris, France). Characterization of the K562/Dox cell line is given in details elsewhere [39]. K562/DoxDR2 cells with decreased expression of ABCB1 were established by stable transfection of K562/Dox cells with a plasmid vector expressing shRNA targeting the *ABCB1* gene [39,40].

K562/ABCG2 cells overexpressing wild type ABCG2 [41] were kindly provided by Prof B. Sarkadi (National Blood Center and Semmelweis University, Budapest, Hungary). Sub-clones K562/ABCG2CL10 and K562/ABCG2CL1 with high and low expression level of ABCG2, respectively, were used in this study. They were established by a single cell cloning by limiting dilution of K562/ABCG2 cells [31].

Resistant cells were cultured under the same conditions as maternal K562 cells.

### 4.3. Determination of Cell Survival and Proliferation

Cell viability and proliferation was determined using the MTT assay as described previously [42].

### 4.4. Analysis of Cell Cycle and Apoptotic Cells

Flow cytometric measurements of DNA content were used to analyze the cell cycle as well as for identification of apoptotic cells (fraction of cells in the sub G1 phase), as described previously [27,43]. Apoptotic cells are expressed as a percentage of cells in sub G1 phase.

### 4.5. Measurement of ROS Production

Intracellular ROS were detected in 2′,7′-dichlorodihydrofluorescein diacetate (H_2_DCF-DA)-loaded cells (Molecular Probe, Leiden, The Netherlands) as described previously [28].

### 4.6. Morphological Analysis of Apoptosis

Fixed cells were stained with Hoechst 33342 Sigma-Aldrich (St. Louis, MO, USA) and morphology of cell nuclei was examined using an Olympus BX60 (Olympus, Hamburg, Germany) fluorescence microscope as described previously [44].

### 4.7. Measurement of Caspase-3 Enzymatic Activity

Caspase-3 enzymatic (DEVDase) activity was measured in cytoplasmic extracts using the fluorescent substrate Ac-DEVD-AMC [45]. It is necessary to note that Ac-DEVD-AMC substrate is efficiently cleaved also by caspase-7. Therefore, we used the term “DEVDase activity” when refering to the cleavage of Ac-DEVD-AMC substrate in the text.

### 4.8. Measurement of Caspase-3 Processing

Western blot analysis was used to directly demonstrate capase-3 processing. Briefly, protein extracts and sample preparation were done as described previously [46]. Caspase-3 processing was determined using Western blot analysis with a polyclonal anti-caspase-3 antibody (1:1000; Cell Signaling Technology, Danvers, MA, USA) recognizing both pro- and active protease forms and polyclonal anti-HSP90 antibody (1:2000; Cell Signaling Technology, Danvers, MA, USA) for detection of reference protein. A horseradish peroxidase-conjugated secondary anti-rabbit antibody (1:2000; Dako, Glostrup, Denmark) in combination with an enhanced chemiluminiscence (ECL; Amersham, Little Chalfont, UK) was used for signal detection.

### 4.9. Western Blot Analysis of Activation of Nrf2 and Stress-Response Pathway

Western blot analysis was used to demonstrate the activation of Nrf2 and its downstream regulated genes, including HO-1 and NQO1 [20]. Protein extracts were done as described previously [46]. Mouse monoclonal anti-Nrf2 antibody (A-10; 1:1000; Santa Cruz Biotechnology, Dallas, TX, USA) was used to determine human ~60 kDa forms of Nrf2. Rabbit polyclonal anti-HO-1 antibody (1:1000; Thermo Fisher Scientific, Waltham, MA, USA) for detection of HO-1 and rabbit polyclonal anti-NQO1 antibody (1:1000; Thermo Fisher Scientific, Waltham, MA, USA) for the detection of HO-1 and NQO1, respectively. Polyclonal anti-HSP90 antibody and polyclonal anti-HSP70 antibody (both 1:2000; Cell Signaling Technology, Danvers, MA, USA) was used for detection of reference protein. Goat anti-rabbit IgG-HRP or goat anti-mouse IgG-HRP secondary antibody (1:2000 or 1:10,000; Dako, Glostrup, Denmark) in combination with an enhanced chemiluminiscence (ECL; Amersham, Little Chalfont, UK) was used for signal detection.

### 4.10. Preparation of Cell Extracts

An optimized acidic extraction of cells after their separation from the culture medium by centrifugation through a layer of silicone oil with a slight modification was used [27,28,47]. Briefly, cells at a density of 5 × 10^5^/mL were incubated in the culture medium (with or without EF-24) for appropriate time periods at 37 °C. Afterwards, cells were centrifuged through silicone oil and cell pellets were extracted as follows: ice cold 5% (*v*/*v*) formic acid was used for GSH and EF-24 analysis; or ice cold 4% (*v*/*v*) formic acid in 40% (*v*/*v*) methanol in water was used for EF-24-GSH and EF-24-NAC adduct analysis. Clarified cell extracts (centrifugation: 40,000× *g* 10 min at 4 °C) were diluted with distilled water and analyzed by liquid chromatography coupled with a low-energy collision tandem mass spectrometer (LC/MS/MS). Alternatively, clarified cell extracts were stored at −80 °C.

### 4.11. High-Performance Liquid Chromatography (HPLC) Analysis of Glutathione (GSH) and Oxidized Glutathione (GSSG)

Quantitative analysis of GSH and GSSG was done using LC/MS/MS during one run with the specific parameters. The chromatographic separations were performed using the high-performance liquid chromatography (HPLC) tower system UltiMate 3000 (Dionex, Germering, Germany), a Polaris C18-A, 5 μm, 250 × 2.0 mm HPLC column (Varian Inc., Lake Forest, CA, USA), and a guard C18, 4.0 × 2.0 mm precolumn (Phenomenex, Torrance, CA, USA). The chromatographic parameters were as follows: the binary gradient of mobile phase A (95% methanol in 0.25% formic acid, *v*/*v*) and B (0.25% formic acid in water, *v*/*v*) from 0–3 min (5 → 23% of solvent A), from 3–4 min (23 → 95% of solvent A), from 4–6 min (95 → 5% of solvent A) and from 6–10 min (5% of solvent A); the flow rate at 0.3 mL/min; the sample injection volume at 5 μL. The API 3200 triple quadrupole mass spectrometer (MDS SCIEX, Concord, ON, Canada) with the TurboIonSpray interface in the positive ion mode was applied for quantification of analytes. The Product Ion Scan mode (GSH: Q1 quadrupole 308.1 amu, Q3 quadrupole at scale 178.95–179.05 amu and GSSG: Q1 quadrupole 613.1 amu, Q3 quadrupole at scale 230.6–231.4 amu) was used. The mass-dependent parametres were optimized: the collision energy and the declustering potential for GSH standard were 17 V and 26 V, and for GSSG standard were 45 V and 51 V, respectively. Ion spray probe parameters were set for GSH and GSSG standards: needle voltage 5500 V and temperature 450 °C. Data were acquired using Analyst^®^ software, ver. 1.5.1 (MDS SCIEX, Concord, ON, Canada).

### 4.12. Determination of EF-24

The HPLC/MS/MS analytical system was described in Section 4.11. A Kinetex HILIC, 2.6 μm, 150 × 2.1 mm column (Phenomenex, Torrance, CA, USA) and a guard Kinetex HILIC, 4.0 × 2.0 mm precolumn (Phenomenex, Torrance, CA, USA) were installed. The binary linear gradient of mobile phase C (95% acetonitrile in 0.25% formic acid, *v*/*v*) and B (0.25% formic acid in water, *v*/*v*) from 0–6 min (95 → 25% of solvent C), from 6–7 min (25 → 95% of solvent C), and from 7–11 min (95% of solvent C) was programmed. The flow rate at 0.15 mL/min and the sample injection volume at 10 μL were set. The mass spectrometer was operated in the multiple-reaction monitoring (MRM) mode. MRM transition mode of 312 > 149 amu (dwell-time = 100 ms) was optimized for quantifying of EF-24. Mass spectrometric parameters were set to the following values: needle voltage, temperature, collision energy, declustering potential and entrance potential at 5500 V, 400 °C, 33.0 V, 46.0 V and 5.0 V, respectively.

### 4.13. Determination of EF-24-GSH and EF-24-NAC Adducts

The HPLC/MS/MS analytical system was described in Section 4.11. For chromatography columns see Section 4.12. The binary gradient of mobile phase C (95% acetonitrile in 0.25% formic acid, *v*/*v*) and B (0.25% formic acid in water, *v*/*v*) from 0–6 min (95 → 75% of solvent C), from 6–7 min (75 → 50% of solvent C), from 7–10 min (50% of solvent C), from 10–11 min (50 → 95% of solvent C), and from 11 → 15 min (95% of solvent C) was used. The flow rate at 0.2 mL/min and the sample injection volume at 10 μL were adjusted. The Product Ion Scan mode was optimized (EF-24–GSH adduct: Q1 quadrupole 619.0 amu, Q3 quadrupole at range 148.8–149.6 amu and EF-24–NAC adduct: Q1 quadrupole 475.4 amu, Q3 quadrupole at range 148.8–149.6 amu). Mass spectrometric parameters were adjusted to the values: needle voltage, temperature, collision energy, declustering potential and entrance potential at 5500 V, 400 °C, 70.0 V, 30.0 V and 5.0 V for EF-24-GSH adduct and 5500 V, 400 °C, 65 V, 35 V and 5.0 V for EF-24-NAC adduct, respectively.

### 4.14. Statistical Analysis

Data are reported as means ± S.D. All statistical analyses were performed using SigmaPlot 11.0 software package (Systat Software Inc., San Jose, CA, USA). Statistical significance of differences was determined by Student’s *t*-tests and one-way ANOVA. *p* values equal to or less than 0.05 were considered significant.

## Figures and Tables

**Table 1:** Effect of *N*-acetylcysteine (NAC) and catalase (CAT) on cell proliferation and viability in EF-24-treated K562 cells.

Cell Treatment	IC_50_
EF-24	0.73 ± 0.11 μM
EF-24 + 2 mM NAC	>20 μM
EF-24 + CAT (50 U/mL)	0.84 ± 0.15 μM

**Table 2:** Effect of ABCB1 or ABCG2 expression on cell proliferation and viability in EF-24 treated K562 cells. ZSQ, zosuquidar trihydrochloride.

	EF-24	EF-24 + ZSQ	EF-24 + Ko143
K562	0.74 ± 0.10 μM	0.71 ± 0.10 μM	0.77 ± 0.12 μM
K562/Dox	0.87 ± 0.15 μM	0.72 ± 0.11 μM	-
K562/DoxDR2	0.75 ± 0.11 μM	0.69 ± 0.10 μM	-
K562/ABCG2CL10	0.71 ± 0.11 μM	-	0.75 ± 0.11 μM
K562/ABCG2CL1	0.72 ± 0.10 μM	-	0.76 ± 0.11 μM
